# Fracture Resistance of Teeth Restored With Different Fiber-Reinforced Composites: A Comparative In Vitro Study

**DOI:** 10.7759/cureus.96424

**Published:** 2025-11-09

**Authors:** Barasha Choudhury, Karan Khaneja, Rishi Manan, Parul Bansal, Digvijay Singh, Nadeem Ashiq

**Affiliations:** 1 Department of Conservative Dentistry and Endodontics, Institute of Dental Studies and Technologies, Modinagar, IND

**Keywords:** composite resins, fiber reinforced materials, fracture, resistance, restoration

## Abstract

Introduction and aim: Restoration of extensively damaged posterior teeth poses a clinical challenge because of the high risk of fractures associated with wide mesio-occluso-distal (MOD) cavities. Conventional nanohybrid composites, although esthetically satisfactory, often fail to reinforce weakened cusps under occlusal stress. Fiber-reinforced composites (FRCs) have emerged as promising alternatives for enhancing flexural strength, fracture toughness, and stress distribution across the restoration-tooth interface. The present in vitro study aimed to compare the fracture resistance of mandibular molars with standardized MOD cavities restored using different FRC systems and conventional composite resins.

Materials and methods: Forty freshly extracted human mandibular molars of similar size and morphology were selected, and standardized MOD cavities were prepared using a high-speed handpiece under water cooling. The samples were randomly divided into four groups (n=10 each). Group 1 (control) was treated with a nanohybrid composite resin (NeoSpectra ST; Charlotte, NC: Dentsply Sirona). Group 2 was restored with FibraFill Dentin (Jičín, Czech Republic: Dentapreg) combined with NeoSpectra ST. Group 3 incorporated Ribbond polyethylene fiber (Seattle, WA: Ribbond Inc.) at the cavity base, whereas Group 4 used EverStick C&B (Tokyo, Japan: GC Corporation) glass fiber beneath the composite restoration. All restorations were performed using standardized etching, bonding, and incremental curing techniques. The specimens were stored in distilled water at 37°C for 24 h before testing. The fracture resistance was evaluated using a Universal Testing Machine (Norwood, MA: Instron) with a crosshead speed of 2 mm/min until fracture occurred. Data were statistically analyzed using one-way analysis of variance (ANOVA) followed by post-hoc Tukey’s test, with the significance level set at p<0.05.

Results: The mean fracture resistance values were highest for EverStick (2759.5±1163.33 N), followed by FibraFill Dentin (2371.1±586.36 N), Ribbond (2057±321.93 N), and the lowest for the control group, NeoSpectra ST (1655.3±345.2 N). The differences between the groups were statistically significant (p=0.008). Pairwise comparisons using the post-hoc Tukey’s test revealed that EverStick showed significantly greater reinforcement than NeoSpectra ST (p=0.006). Other comparisons, including NeoSpectra ST versus FibraFill Dentin, NeoSpectra ST versus Ribbond, FibraFill Dentin versus Ribbond, FibraFill Dentin versus EverStick, and Ribbond versus EverStick, did not show statistically significant differences (p>0.05).

Conclusion: Within the limitations of this in vitro study, it can be concluded that the incorporation of FRCs considerably improves the fracture resistance of teeth with large MOD cavities compared with conventional composites. Among the tested materials, EverStick glass fiber exhibited the best performance, followed by FibraFill Dentin and Ribbond.

## Introduction

Dental caries continues to be one of the most widespread chronic diseases globally, impacting oral health across all age groups and necessitating innovative restorative interventions. Despite progress in preventive measures and public education, the incidence of tooth decay persists, driving the demand for durable and esthetically pleasing restorative materials [1]. Composite resins have become the material of choice for both anterior and posterior restorations owing to their excellent esthetics, conservative tooth preparation, and affordability [2]. However, in structurally compromised teeth, particularly those with extensive class II mesio-occluso-distal (MOD) cavities, these restorations face significant challenges, including heightened susceptibility to fractures under occlusal stresses [3]. The loss of tooth structure during cavity preparation weakens overall integrity, with marginal ridges playing a pivotal role in maintaining stability. Wide MOD cavities amplify stress concentrations, leading to horizontal forces that can fracture cavity walls and underscoring the need for enhanced reinforcement strategies [3-5].

To address these limitations, advancements in composite technology have introduced nanofillers and fiber-reinforced composites (FRCs), which improve mechanical properties, such as flexural strength, fracture toughness, and resistance to crack propagation [6]. FRCs integrate fibers into the resin matrix, creating a reinforced structure that distributes occlusal loads more evenly and minimizes premature failure. This innovation has revolutionized restorative dentistry, offering superior durability in high-stress posterior regions where traditional composites may falter owing to polymerization shrinkage, secondary caries, or marginal breakdown [6,7]. Research highlights that factors, such as cavity size, fiber type, and placement technique, critically influence restoration performance, with larger cavities exacerbating fracture risk [8,9]. Over the past two decades, materials, such as ultra-high-molecular-weight polyethylene fibers, have been employed to prevent crack formation and enhance longevity by embedding them in flowable composites or axial walls [10].

Studies evaluating FRCs have demonstrated their efficacy in improving compressive strength and overall resilience, although variability in testing conditions, such as mounting methods and loading speeds, can affect outcomes [5-10]. For instance, short glass fiber-reinforced resin composites and polyethylene fiber-reinforced resin composites have shown promising results in reinforcing weakened cusps, whereas nanohybrid composites provide balanced esthetics and handling [6,10].

This study aimed to compare the fracture resistance of molars with class II MOD cavities restored with different FRCs as follows: Neospectra ST (Charlotte, NC: Dentsply Sirona) (control), FibraFill Dentin (Jičín, Czech Republic: Dentapreg), Ribbond (Seattle, WA: Ribbond Inc.), and EverStick (Tokyo, Japan: GC Corporation). The objectives included preparing standardized MOD cavities in extracted molars, restoring them with the specified materials, evaluating fracture resistance, and statistically analyzing differences to determine the most effective reinforcement for posterior restorations.

## Materials and methods

This in vitro experimental study was conducted at the Department of Conservative Dentistry and Endodontics, Institute of Dental Studies and Technologies (IDST), Modinagar, Uttar Pradesh, India. The duration of the study spanned over three months, including phases of sample collection, cavity preparation, restoration, and testing. Ethical clearance was obtained from the Institutional Ethical Committee (approval number: IDST/IEC/2022-25/04). All extracted teeth used in this study were collected from the Department of Oral and Maxillofacial Surgery after obtaining written informed consent from the patients.

Sample size estimation

G*Power analysis version 3.1.9.7 (Düsseldorf, Germany: Heinrich Heine University Düsseldorf) was used to estimate the appropriate sample size for this study. Based on an effect size of 0.55 (and a previous reference comparing fracture resistance between composite systems), it was determined that a minimum of 40 samples would provide robust comparative results [11]. For the present study, with four experimental groups, a priori F-test (fixed effect, omnibus) was conducted using a power of 80% and an alpha error of 5%. The analysis indicated that 40 samples (10 per group) were required to achieve reliable statistical outcomes.

Inclusion and exclusion criteria

Forty freshly extracted permanent human mandibular molars of similar sizes and morphologies were selected. Teeth with intact crowns, mature apices, no evidence of cracks, caries, restorations, abrasions, or structural anomalies were included. Teeth with open apices, hypoplastic defects, or previous restorations were excluded to ensure uniformity. All teeth were thoroughly cleaned using an ultrasonic scaler (Guilin, China: Woodpecker) to remove calculus and debris. They were then disinfected in a 0.1% thymol solution and stored in distilled water at room temperature until use to prevent dehydration.

Procedure

Each tooth was mounted vertically in an acrylic resin block (DPI Self Cure Acrylic; Mumbai, India: Dental Products of India Ltd.) with the long axis perpendicular to the base, leaving 1.5 mm of the root exposed below the cementoenamel junction to simulate alveolar bone level. Standardized MOD cavities were prepared using a high-speed air-rotor handpiece (Tokyo, Japan: NSK/Nakanishi Inc.) under water cooling using a 245-tungsten carbide straight fissure bur (Lakewood, NJ: SS White Dental). Each bur was replaced after every five preparations to maintain the cutting efficiency and standardization. The cavity dimensions were standardized at 2 mm pulpal width, 1.5 mm gingival width, and 2 mm buccolingual width, maintaining parallel axial walls and a 90° cavosurface margin. The cavity dimensions were verified using a digital Vernier caliper (Kawasaki, Japan: Mitutoyo Corporation).

To ensure reproducibility, all cavity preparations were performed by a single calibrated operator. Calibration was achieved by preparing 10 pilot samples and re-measuring cavity dimensions until intra-operator reliability exceeded 0.9 using the intra-class correlation coefficient (ICC). This ensured consistency in the cavity size, angulation, and depth across all specimens.

The 40 specimens were randomly divided into four groups of 10 each, based on the restorative material used. Group 1 (control) was treated with a nanohybrid composite resin, NeoSpectra ST (Charlotte, NC: Dentsply Sirona). Group 2 was restored using FibraFill Dentin (Jičín, Czech Republic: Dentapreg) at the cavity base, followed by the NeoSpectra ST composite. Group 3 incorporated Ribbond polyethylene fibers (Seattle, WA: Ribbond Inc.) at the base of the cavity before composite placement, and Group 4 used EverStick C&B fibers (Tokyo, Japan: GC Corporation) beneath the composite restoration. Fiber-reinforced materials were positioned according to the manufacturer's instructions. Ribbond fibers were placed at the cavity base and adapted to the internal walls before the first composite increment, EverStick C&B fibers were positioned horizontally along the cavity floor beneath the composite, and FibraFill Dentin fibers were incorporated as a pre-impregnated layer at the dentin interface prior to incremental composite buildup.

The adhesive protocol was standardized across all groups. A 37% phosphoric acid etchant (Charlotte, NC: Dentsply Sirona) was applied to the enamel and dentin for 15 s, rinsed for 10 s, and gently air-dried to achieve a moist surface. A universal adhesive bonding agent, Prime&Bond active (Charlotte, NC: Dentsply Sirona), was applied with a microbrush for 20 s, air-dried for 5 s, and light-cured for 10 s using a visible light-curing unit (Bluephase N; Schaan, Liechtenstein: Ivoclar Vivadent) with an intensity of 1200 mW/cm². The restorative materials were placed incrementally in 2 mm layers, and each layer was light-cured for 20 s. In the fiber-reinforced groups, the fibers were positioned according to the manufacturers’ recommendations (either adapted at the cavity base or embedded within the first composite increment) to optimize reinforcement.

Following restoration, all specimens were finished using an acrylic trimming bur (Au, Switzerland: Edenta AG) and polished using Sof-Lex discs (St. Paul, MN: 3M ESPE). The restored samples were stored in distilled water at 37°C for 24 h to allow post-curing polymerization and hydration, simulating oral conditions. The operator performing cavity preparation and restoration was not involved in fracture testing or data analysis. The investigator conducting the fracture resistance testing and statistical analysis was blinded to group allocation to minimize bias.

The fracture resistance of all samples was evaluated using a Universal Testing Machine (Norwood, MA: Instron). Each tooth was positioned on a metal jig so that the load was applied vertically to the center of the occlusal surface at a 90° angle to the long axis using a stainless steel ball with a diameter of 5 mm. The load was applied at a crosshead speed of 2 mm/min until a fracture occurred. The maximum load required to fracture each specimen was recorded in Newtons (N) and statistically analyzed.

Statistical analysis

Data analysis was conducted using Statistical Package for Social Sciences (SPSS) software version 23 (Armonk, NY: IBM Corp.). The data were confirmed to be normally distributed using the Shapiro-Wilk test. The fracture resistance values of the study groups are presented as mean and standard deviation, along with a confidence interval of 95%. The groups were compared using one-way analysis of variance (ANOVA), followed by post-hoc Tukey’s test for pairwise group comparisons. A p-value less than 0.05 was considered significant for all comparisons.

## Results

Descriptive analysis of the study groups showed variability in the measured fracture resistance of the teeth across the four groups. NeoSpectra ST had a mean value of fracture resistance of 1655.3±345.2 N. FibraFill Dentin showed the highest mean fracture resistance of 2371.1±586.36 N. Ribbond presented a moderate mean of 2057±321.93 N. EverStick had the widest range of 1227-4289 and the highest variability (mean: 2759.5±1163.33 N), as shown in Table 1.

**Table 1:** Descriptive analysis of study groups for fracture resistance (Newtons). Samples are presented as frequency (n) and percentage (%), whereas data are presented as mean and standard deviation (SD).

Groups	n (%)	Minimum	Maximum	95% CI for mean	Mean±SD
NeoSpectra ST (control)	10 (25%)	1156	2134	1408.36-1902.24	1655.30±345.20
FibraFill Dentin	10 (25%)	1609	3121	1951.64-2790.56	2371.10±586.36
Ribbond	10 (25%)	1661	2635	1826.71-2287.29	2057.00±321.93
EverStick	10 (25%)	1227	4289	1927.30-3591.70	2759.50±1163.33

A one-way ANOVA comparing the fracture resistance of different composite materials showed a statistically significant difference among groups (p=0.008), with a moderate effect size of 0.28. EverStick exhibited the highest mean fracture resistance (2759.5±1163.33 N), followed by FibraFill Dentin (2371.1±586.36 N) and Ribbond (2057±321.93 N). NeoSpectra ST had the lowest mean resistance (1655.3±345.2 N). These results suggest that the EverStick and FibraFill Dentin composites provided superior fracture resistance compared with the other materials studied (Table 2).

**Table 2:** Comparison of fracture resistance (Newtons) between groups using one-way analysis of variance (ANOVA). *P<0.05 denotes statistical significance using one-way ANOVA test. Data are presented as mean and standard deviation (SD).

Groups	Mean±SD	F stats	p-Value	Effect size
NeoSpectra ST (control)	1655.30±345.20	4.58	0.008*	0.28
FibraFill Dentin	2371.10±586.36			
Ribbond	2057.00±321.93			
EverStick	2759.50±1163.33			

Post-hoc Tukey’s test for pairwise group comparisons revealed a significant difference only between NeoSpectra ST and EverStick (mean difference = -1104.2 N, p=0.006). Other comparisons, including NeoSpectra ST versus FibraFill Dentin, NeoSpectra ST versus Ribbond, FibraFill Dentin versus Ribbond, FibraFill Dentin versus EverStick, and Ribbond versus EverStick, did not show statistically significant differences (p>0.05). The significant difference between NeoSpectra ST and EverStick suggested that EverStick showed considerably higher fracture resistance than NeoSpectra ST, highlighting its potential clinical advantage (Table 3).

**Table 3:** Pairwise comparison of fracture resistance (Newtons) of study groups using post-hoc Tukey’s test. *P<0.05 denotes statistical significance using post-hoc Tukey’s test.

Paired groups	Mean difference (Newtons)	T stats	p-Value	95% CI lower limit	95% CI upper limit
NeoSpectra ST - FibraFill Dentin	-715.8	-2.31	0.160	-1580.85	149.25
NeoSpectra ST - Ribbond	-401.7	-1.30	1.00	-1266.75	463.35
NeoSpectra ST - EverStick	-1104.2	-3.56	0.006*	-1969.25	-239.15
FibraFill Dentin - Ribbond	314.1	1.01	1.00	-550.95	1179.15
FibraFill Dentin - EverStick	-388.4	-1.25	1.00	-1253.45	476.65
Ribbond - EverStick	-702.5	-2.27	0.177	-1567.55	162.55

## Discussion

The results of this in vitro study revealed significant differences in fracture resistance among the four groups of molars with standardized class II MOD cavities. The control group exhibited the lowest mean fracture resistance, whereas the FRC groups showed progressively higher values. These findings align with the hypothesis that FRCs enhance the structural integrity of weakened posterior teeth [7], and glass fiber-based systems, such as EverStick, demonstrate superior performance [12].

The enhanced fracture resistance in the FRC groups can be attributed to the role of the fibers in stress distribution and crack deflection. Fibers act as internal reinforcements, bridging microcracks and dissipating occlusal forces across the restoration-tooth interface, thereby reducing cuspal flexure and preventing catastrophic failures [7,12]. EverStick, a unidirectional glass fiber impregnated with polymethyl methacrylate, likely outperformed the others owing to its high tensile strength (approximately 800-1000 MPa) and optimal bonding to the composite matrix, allowing for better load transfer. This finding is consistent with previous in vitro studies evaluating EverStick in MOD cavities, which have shown that it increases fracture loads by 30-50% compared to non-reinforced composites. For instance, Garoushi et al. reported similar improvements in premolars restored with EverStick, attributing the gains to the anisotropic properties of the fiber that mimic the modulus of elasticity (18-20 gigapascals {GPa}) [13]. Lin et al. reported an increase in the flexural strength in the longitudinal and transverse directions using unidirectional glass fiber composites [14].

Ribbond, a polyethylene fiber with a leno-weave structure, also showed robust reinforcement, likely owing to its plasma-treated surface, which enhances adhesion and its ability to absorb energy through plastic deformation. Belli et al. found that Ribbond increased the fracture resistance in endodontically treated molars by 25-40%, supporting our observations, although its lower modulus (2-3 GPa) may explain why it underperformed relative to glass fibers such as EverStick [15]. Similar findings were reported in a previous study [16].

FibraFill Dentin, a pre-impregnated glass fiber bundle designed for bulk placement, provided moderate reinforcement after EverStick, possibly limited by its shorter fiber length and less uniform distribution compared with EverStick’s continuous fibers. This aligns with Dentapreg's manufacturer’s claims of improved flexural strength, but our results suggest that it is less effective in large cavities than woven or unidirectional fibers [17]. Previous studies reported an increase in failure rates when pre-impregnated glass fibers were used with bisphenol A glycidyl methacrylate (Bis-GMA)-based composites, and therefore recommended the use of an intermediate adhesive resin layer [18,19].

The lower resistance of the control group reflects the inherent limitations of nanohybrid composites, such as NeoSpectra ST, which, despite low shrinkage (1.5-2%), lacks a fiber network to counteract tensile stresses at the cavity floor. This is corroborated by Galyan et al., who reported that nanocomposites restored with a single fiber showed a maximum fracture resistance similar to that of a natural tooth, compared to nanocomposites without fiber reinforcement [20].

These results are based on evidence favoring FRCs over conventional composites for high-stress restorations. A meta-analysis by Escobar et al. of 24 in vitro studies confirmed that FRCs elevate fracture resistance compared to non-fiber-reinforced restorations, particularly in molars, owing to enhanced energy absorption [7]. The study by Karadağ and Erdal found that polyethylene fiber (Ribbond) did not significantly enhance fracture resistance in large MOD cavities, contrasting with our findings, where Ribbond and other FRCs (EverStick, FibraFill Dentin) significantly improved fracture resistance compared to NeoSpectra ST [21]. This discrepancy may arise from differences in cavity design (e.g., depth, width, or fiber placement), adhesive protocols, or testing conditions (e.g., load application or thermocycling).

The clinical implications of these findings are substantial. For practitioners treating large MOD lesions in molars, incorporating FRCs, such as EverStick or Ribbond, could reduce post-operative fractures, extend restoration longevity, and minimize the need for invasive interventions, such as crowns. This is particularly relevant in conservative dentistry, as it preserves tooth structure while enhancing durability under masticatory forces (up to 800 N in molars). EverStick's adaptability for chairside customization makes it ideal for routine use, potentially improving outcomes in patients with bruxism or heavy occlusions. However, clinicians should prioritize proper fiber placement at the cavity base to maximize reinforcement, as per manufacturer guidelines, and combine it with universal adhesives for optimal bonding.

The limitations of this study must be acknowledged. As an in vitro study, it lacks simulation of oral dynamics, such as thermocycling, pH fluctuations, or fatigue loading, which could degrade materials over time; therefore, future research should incorporate 10,000+ cycles to mimic one-year clinical service. The sample size, while powered at 80%, may not capture variability in tooth morphology; larger cohorts or finite element analysis could refine the predictions. Storage in distilled water for 24 h is simplistic, potentially underestimating the effects of hydrolysis on fiber-matrix bonds. Extracted teeth may not fully represent vital dentin, and vertical loading at 2 mm/min differs from intra-oral multidirectional forces. Additionally, the absence of periodontal ligament simulation may have influenced stress distribution patterns, potentially differing from clinical conditions. Cyclic fatigue loading and long-term aging effects were not assessed, which could alter fracture resistance over time. Although all cavity preparations were performed by a single calibrated operator to minimize variability, minor residual differences in technique cannot be entirely excluded.

## Conclusions

Within the limitations of this in vitro study, it can be concluded that the incorporation of FRCs, particularly EverStick, significantly enhanced the fracture resistance of teeth with extensive MOD cavities compared to that of the non-fiber-reinforced NeoSpectra ST. Although FibraFill Dentin and Ribbond groups exhibited higher mean fracture resistance values, these differences were not statistically significant. The findings suggest that fiber reinforcement, particularly using unidirectional glass fibers, can enhance the mechanical performance of restorations in standardized MOD cavities under controlled conditions. However, these results cannot be extrapolated to clinical longevity or to the replacement of full-coverage restorations without further long-term and fatigue-based evaluations.
